# Methylated RNA Immunoprecipitation Sequencing Reveals the m^6^A Landscape in Oral Squamous Cell Carcinoma

**DOI:** 10.1155/2022/7277583

**Published:** 2022-07-15

**Authors:** Xi Wang, Jie Wu, Leyu Zhang, Wei Zhao, Jiayin Deng

**Affiliations:** The School and Hospital of Stomatology, Tianjin Medical University, Tianjin 300070, China

## Abstract

N6-methyladenosine (m^6^A) is the most common epigenetic modification existing in eukaryocyte transcripts. However, genes related to m^6^A modification in oral squamous cell carcinoma (OSCC) are still unclear. Here, methylated RNA immunoprecipitation sequencing (MeRIP-Seq) was performed to map the m6A landscape in OSCC and corresponding controls. The m^6^A peaks are always distributed in the junction of the 3′-untranslated regions (3′-UTRs) and the coding sequences (CDS) of mRNAs, as well as the entire genome of long noncoding RNA (lncRNA). Furthermore, enrichment analysis showed that differentially methylated genes were significantly enriched in NF-kappa B signaling pathway, Hedgehog signaling pathway, etc. In summary, our findings reveal the landscape of m^6^A modification on mRNAs and lncRNAs in OSCC, which may provide key clues for the precision-targeted therapy of OSCC.

## 1. Introduction

Similar to histones and DNAs, messenger RNAs (mRNAs) and noncoding RNAs (ncRNAs) can be likewise chemically modified. In fact, all biological macromolecules are modified in an extremely specific and efficient way to achieve the functions for which they are designed. In addition, RNA methylation and demethylation afflict primary microRNAs (pri-miRNAs), ribosomal RNAs (rRNAs), transfer RNAs (tRNAs), and enhancer RNA (eRNAs) (1, 2). Nowadays, RNA modifications and their functions are increasingly becoming familiar and understood due to the dramatic development of high-throughput sequencing technologies. Meanwhile, these RNAs can function as competing endogenous RNAs (ceRNAs) or natural miRNA sponges to form long noncoding RNA- (lncRNA-) miRNA-mRNA ceRNA networks and competitively bind to miRNAs for communication and coregulation. Therefore, they exert enormous impacts on gene regulatory networks and human diseases (3).

With deeper investigations of RNA modifications, it has been discovered that there are over 150 dissimilar methods, including N6-methyladenosine (m^6^A), 5­methylcytosine (m^5^C) in RNA, N^1^­methyladenosine (m^1^A), 7­methylguanosine (m^7^G), pseudouridine, and uridylation (U-tail) (4–6). Current research has reported m^6^A as the most common and conserved epigenetic modification. With the participation of m^6^A methyltransferases (writers), demethylases (erasers), and binding proteins (readers), m^6^A is engaged in cell proliferation, differentiation, invasion, migration, metabolism, apoptosis, and pyroptosis to control cell functions, including embryonic development (7, 8), spermatogenesis (9), and tumor growth or inhibition (10, 11), by manipulating diverse biological processes, such as RNA stability, splicing, exportation, localization, translation, export, and pri-miRNA processing.

As reported, m^6^AVar, a comprehensive database, involves various m^6^A-associated changes that have an unrecognized possibility to affect the m^6^A modification. M^6^AVar can be regarded as an extremely critical auxiliary and research recourse to reveal the interactions of m^6^A-associated macromolecules with conditions and disorders. In this database, methylated RNA immunoprecipitation with next generation sequencing (MeRIP-Seq) is applied to find m^6^A-associated mutations and fill them in (12).

In the meantime, MeRIP-Seq has been employed in several studies to investigate the mechanisms underpinning m^6^A in different diseases. Liu et al. used MeRIP-Seq and MeRIP-PCR to find that METTL3 could promote OSCC proliferation and metastasis through the m6A modification in the 3′ UTR of BMI1 mRNA (13). Zhang et al. observed a consensus RRACH motif approach in both preferential distributions of m^6^A peaks near the stop codon and m^6^A methylome on zebrafish using MeRIP-Seq and m^6^A individual-nucleotide-resolution cross-linking and immunoprecipitation with sequencing. The downregulation of m^6^A is related to the halt of hematopoietic stem/progenitor cell (HSPC) emergence (7). Moreover, our preliminary study found that methyltransferase-like protein 3 (METTL3) increased the stability of c-Myc by establishing m^6^A modification on the 3′ untranslated regions (3′UTRs; near the stop codon) of c-Myc to influence oral squamous cell carcinoma (OSCC) cell invasion, migration, and proliferation, which revealed the relationship with the negative prognosis and tumorigenesis of OSCC patients (14).

However, the function of m^6^A modification in OSCC still lacks comprehensive studies and adequate understanding. In this context, our research is aimed at thoroughly investigating the effects of m^6^A modification on OSCC through MeRIP-Seq and analyzing the differences between the normal function and tumor tissues. Human aortic endothelial cells (HAEC) (15) were used as control to compare with human tongue phosphorous carcinoma cells (SCC25). Afterward, directions for further research and the application of m^6^A were found in the area of microarrays, probes, and screening.

## 2. Results

### 2.1. M^6^A-Specific Peak Sequence Motif Analysis

**Figure 1:**
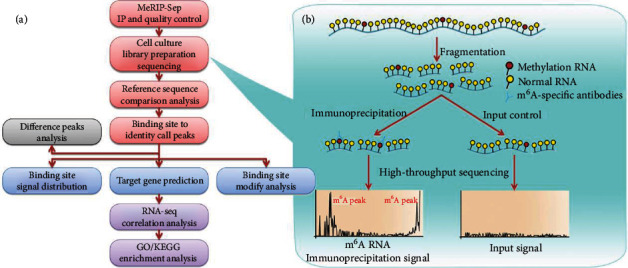
MeRIP-Sep and peak calling analysis. (a) As shown in the flowchart, each group comprised two types of sample RNAs, that is, immunoprecipitation (IP) samples and input control samples, which were fragmented into ~100 nucleotides and immunoprecipitated with m^6^A-specific antibodies. (b) Following the library construction, high-throughput sequencing, and bioinformatics analysis, the location of m^6^A in the whole transcriptome could be provided. Peak calling referred to the use of certain statistical methods to detect the area (called peak) where the reads were remarkably enriched in RNA, as a candidate for m^6^A modification sites. After the peak was obtained, peak sequence motif and peak annotation analyses were performed in the peak to further explore the direction of interest.

**Figure 2:**
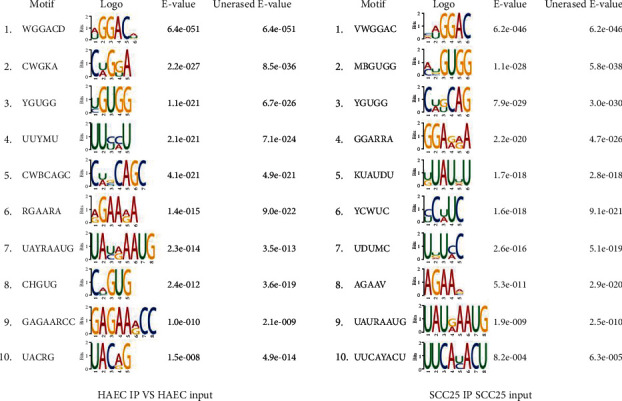
M^6^A-specific peak sequence motif analysis. In genetics, a sequence motif is a widespread nucleotide or amino acid sequence pattern. In the apparent data analysis, a specific base sequence with a high affinity to certain proteins was generally defined as a motif. M^6^A-specific motif meant a specific base sequence RNA which was modified by m^6^A. The name of the motif was created using the IUPAC codes for nucleotides which had dissimilar letters to express the 15 probable assemblies. The name itself was an expression of the motif although the position weight matrix was not diametrically synonymous as it was produced from the sites that were found to match the letters given in the name. The *E*-value was the enrichment *P* value multiplied by the number of candidate motifs tested. The enrichment *P* value was calculated using Fisher's exact test for the enrichment of the motif in the positive sequences. The counts used in Fisher's exact test were made after the erasing of the sites that matched the previously found motifs. Unerased *E*-value was the *E*-value of the motif calculated without erasing the sites of the previously found motifs.

A motif is described as a specific base sequence that has a high affinity for certain proteins in the apparent data analysis. In addition, a sequence motif is an extensive nucleotide or amino acid sequence pattern in genetics. M^6^A-specific motif implies a specific base sequence RNA that is modified by m^6^A. A series of IUPAC codes were yielded after the sequence of cell culture, library preparation, and MeRIP-Seq (Figures 1(a) and 1(b)). The name of the motif was derived from the IUPAC codes for nucleotides which had different letters to represent 15 probable assemblies. Although the position weight matrix was not completely synonymous, the name itself was an expression of the motif as the motif was generated from the sites that were discovered to match the letters given in the name. For the top 5000 peaks, the base sequences were extracted by widening 100 bp from the upstream and downstream directions, respectively, and then, the MEME software was utilized to predict the motif of these base sequences. Comparisons between the two groups (HAEC IP vs. HAEC input and SCC25 IP vs. SCC25 input) indicated that the nucleotide acid sequence GGAC was a frequently appearing motif. In the peaks identified by m^6^A-Seq data with good quality, “GGAC” predominantly arose at the top of the motif results (Figure 2).

### 2.2. Analysis of Differences between the Two Groups

In order to study the differences in m^6^A sites between the two groups, we processed the peaks between groups as follows: (A) the peaks in the group (the common region and the intersection part were obtained) were integrated into a whole peak file; (B) the peaks between the groups (the union region and the union part was used) were merged into one peak file; (C) the signal value of each sample was counted in the peak interval merged between the groups; (D) as per the signal value of the two groups (SCC25 vs. HAEC), their respective IP samples were compared with the corresponding input samples to obtain the *E*-score value (enrichment score) and calculate the differences in the *E*-score value between the two groups. The difference peaks were determined with the criteria of the mean *E*-score value of the two groups greater than 6 and the multiple log_2_ values of the *E*-score difference greater than 1 (or less than -1).

**Figure 3:**
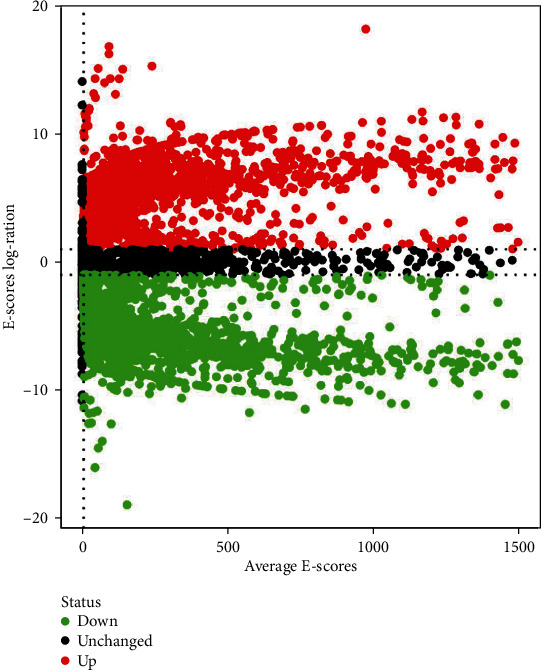
Functional area peak distribution. By exhibiting the involved upregulated and downregulated transcripts, the volcano plot demonstrated the distinct expression of transcripts in the MeRIP-Seq analysis. The abscissa represented the mean *E*-scores of the two groups, and the ordinate marked the multiple log_2_ values of *E*-scores in the two groups.

By exhibiting the involved upregulated and downregulated transcripts, the volcano plot revealed the distinct expression of transcripts in the MeRIP-Seq analysis. The abscissa represented the mean *E*-scores of the two groups, while the ordinate indicated the multiple log_2_ values of the *E*-score difference in the two groups (Figure 3).

### 2.3. Peak Frequency Distribution in the Transcript Area

Theoretically, the m^6^A peak of mRNAs should be enriched near the 3′UTR. A more intuitive understanding of the data characteristics can be obtained based on the distribution of peaks in the transcript area. The R software package Guitar was applied to calculate the frequency of peaks falling on each locus in mRNA and lncRNA transcript regions for each sample, followed by the plotting of a frequency distribution map. The abscissa stood for the 5′UTR, coding sequences (CDS), the 3′UTR of mRNA transcripts, and the entire interval of lncRNAs, whereas the ordinate marked the frequency of peaks falling on these regions.

**Figure 4:**
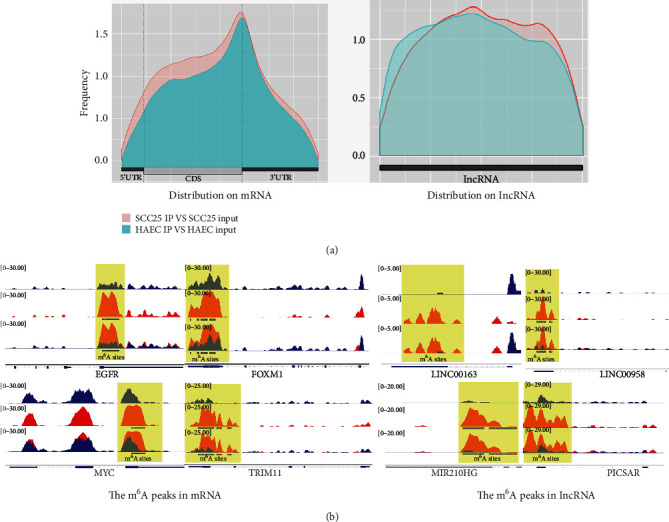
Peak frequency distribution in the transcript area. (a) In theory, the m^6^A peak of mRNAs should be enriched near the 3′UTR. According to the distribution of peaks in the transcript area, the data characteristics can be understood more intuitively. The R software package Guitar was utilized to calculate the frequency of peaks falling on each site in the mRNA and lncRNA transcript regions for each sample, after which, the frequency distribution map was drawn. The abscissa stood for the 5′UTR, CDS, and 3′UTR regions on the mRNA transcript and the entire interval of lncRNA, and the ordinate indicated the frequency of peak falling on these regions. (b) The m^6^A sites were analyzed by the Integrative Genomics Viewer (IGV) tool.

The Integrative Genomics Viewer (IGV) tool was applied to discover the m^6^A sites. The m^6^A peak distribution on mRNAs was compatible with the theory. From peak frequency distribution, it was noted that the m^6^A peaks were always decorated in the head of 3′UTRs and the tail of CDS of target functional mRNAs. While the distribution on lncRNAs suggested that the m^6^A modification on lncRNAs showed no obvious peak and could appear in various parts of lncRNAs (Figure 4(a)). These regions controlled biological activities by methylation or demethylation. Such decoration was found in EGFR, FOXM1, MYC, and TRIM11 of mRNAs and LINC00163, LINC00958, MIR210HG, and PICSAR of lncRNAs (Figure 4(b)).

### 2.4. Peak Distribution in the Functional Area

**Figure 5:**
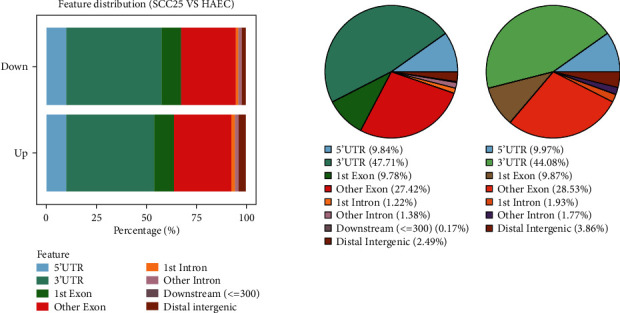
Functional area peak distribution. Metagene profile demonstrated the m^6^A allocation in 5′UTR, CDS, stop codon, and 3′UTR. After the calculation of the peak distribution in the functional area covered by the peak annotation analysis results, the R software package ChIPseeker was applied to plot the statistical results into a histogram where all samples were compared together and plot each sample into a pie chart for separate viewing. In the histogram, the ordinate represented the sample, and the abscissa stood for the ratio of peaks in each functional area. The pie chart exhibited the exact percentage of peaks in each functional area. 5′UTR: the ratio of peaks falling within the 5′UTR; 3′UTR: the ratio of peaks falling within the 3′UTR; 1st exon: the ratio of peaks falling within the first exon of the transcript; other exon: the ratio of peaks falling within other exons except for the first exon of the transcript; 1st intron: the ratio of peaks falling within the first intron of the transcript; other intron: the ratio of peaks falling within other introns except for the first intron of the transcript; distal intergenic: the ratio of peaks falling in the intergenic region.

The R software package ChIPseeker was adopted to retrieve the genes covered by each peak and annotate the functional areas covered by it, also called peak annotation analysis, followed by the calculation of the peak distribution in the functional area covered. Following that, the same R software package was utilized to plot the statistical results into a histogram where all samples were compared together and plot each sample as a pie chart for separate viewing. In the histogram, the ordinate indicated the sample, and the abscissa stood for the ratio of peaks in each functional area. The pie charts displayed the exact percentage of peaks in each functional area. As reflected in the volcano plot, the proportions of different functional areas in upregulated and downregulated transcripts were similar to their corresponding transcripts (Figure 5).

### 2.5. Gene Ontology (GO) Enrichment Analysis

GO is an international standardized gene function classification system. It was created mainly to resolve the confusion in the definition of the same gene in different databases and the confusion in the functional definition of the same gene in different species. GO standard vocabularies consist of three aspects: biological process (BP), molecular function (MF), and cellular component (CC) (16).

**Figure 6:**
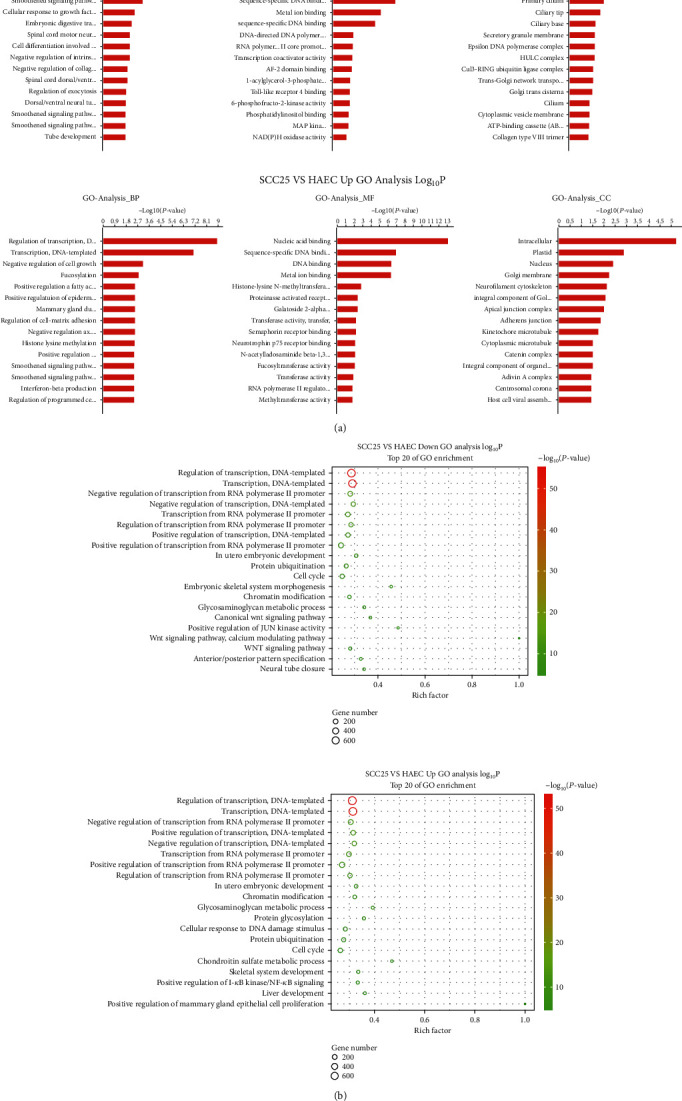
Gene Ontology (GO) enrichment analysis. (a) GO enrichment analysis aimed to obtain significantly enriched GO entries to show gene functions that might be regulated by transcription factors. Each peak-neighboring gene was annotated with GO to harvest all of its GO function entries, and all peak-neighboring genes were assigned to different levels of MF (molecular function), BP (biological process), and CC (cellular component). In the GO functional classification, Fisher's exact test was adopted to calculate the significance level of gene enrichment in each GO classification to screen out the GO classifications that were significantly enriched in the peak-neighboring genes. The top 15 items of GO items that were significantly enriched in the peak-neighboring genes were selected as a histogram, and the significance threshold was *P* value < 0.05. The horizontal axis histogram was taken as an example to display the GO enrichment analysis of all peak-neighboring genes. On the leftmost was BP, in the middle was MF, and on the rightmost was CC. The horizontal axis of each graph was -log10 (*P* value), and the vertical axis was the name of the GO items. (b) The results of the GO enrichment analysis of peak-neighboring genes were presented in a bubble chart format, with the top 20 GO entries that were significantly enriched in peak-neighboring genes displayed. In the figure, the ordinate indicated the GO entry, and the abscissa marked the rich factor. The size of the dot in the figure indicated the number of peak-neighboring genes enriched on the GO entry, and the color illustrated the significance *P* value of the GO entry. The smaller the *P* value, the smaller the enrichment and the more significant the set. The degree of the GO enrichment was measured using the rich factor, *P* value, and the number of genes enriched in this GO entry. Among them, the rich factor referred to the ratio of the number of peak-neighboring genes enriched in the GO entry to the number of all genes annotated to the GO entry. The greater the rich factor, the greater the enrichment.

We first downloaded GO annotations from NCBI (http://www.ncbi.nlm.nih.gov/), UniProt (http://www.uniprot.org/), and the Gene Ontology (https://www.geneontology.org/). GO annotations were performed for all neighboring genes of each peak to collect, and GO function items were classified into MF, BP, and CC levels. Significant GO items were identified using Fisher's exact test to find the gene functions that might be orchestrated or mediated by transcription factors. The false discovery rate (FDR) was applied to correct the *P* values (Figures 6(a) and 6(b)**)**.

Among the downregulated genes, the major BP included “regulation of transcription, DNA-templated,” “smoothened signaling pathway involved in dorsal/ventral neural tube patterning,” and “cellular response to growth factor stimulus;” the major MF were “nucleic acid binding,” “DNA binding,” and “sequence-specific DNA binding;” while “nucleus,” “intracellular,” and “primary cilium” were selected as the key CC. Among the upregulated genes, the major BPs are “regulation of transcription, DNA-templated,” “negative regulation of cell growth,” and “fucosylation;” the major MFs are “nucleic acid binding,” “sequence-specific DNA binding,” and “metal ion binding;” as well as the major CCs are “intracellular,” “plastid,” and “nucleus.”

### 2.6. Pathway Enrichment Analysis

The pathway enrichment analysis was conducted to investigate the metabolic pathways that were obviously enriched in the peak-neighboring genes based on the Kyoto Encyclopedia of Genes and Genomes (KEGG) database. All of the peak-neighboring genes were assigned to different metabolic pathways (17).

**Figure 7:**
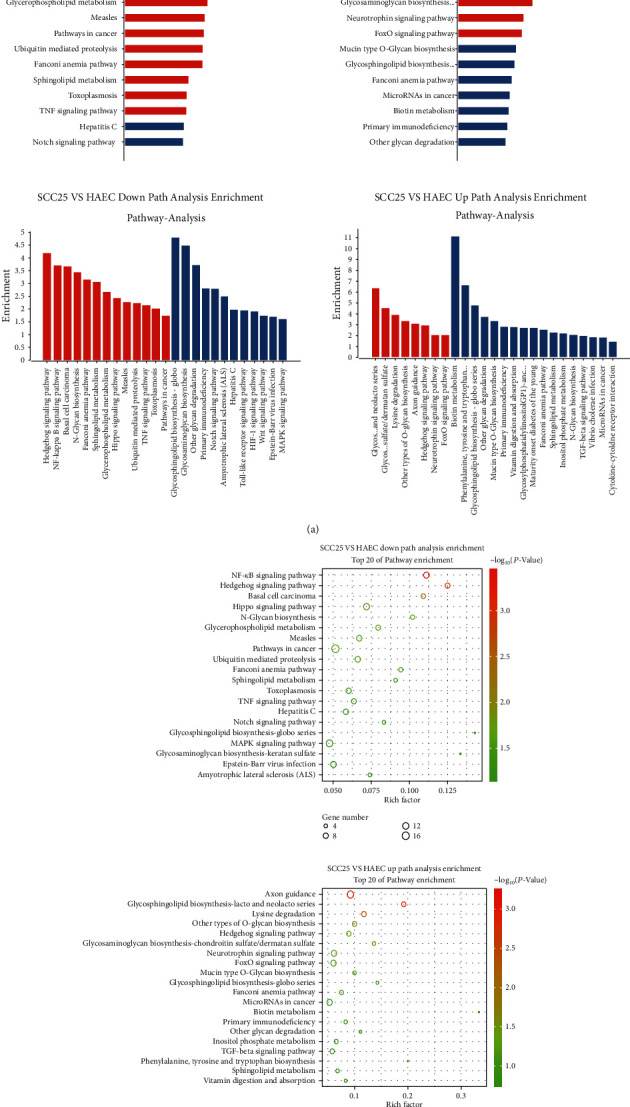
Pathway enrichment analysis. (a) The pathway enrichment analysis was performed to find metabolic pathways that were significantly enriched in peak-neighboring genes based on the KEGG database, thereby investigating metabolic pathways that might be orchestrated by transcription factors and that might be mediated by transcription factors at different developmental stages and cell states. The pathway enrichment analysis can directly reflect the effect of genes on phenotypes and maximize the relationship between significantly enriched metabolic pathways and phenotypes. The selected peak-neighboring genes were annotated based on the KEGG database to obtain all of the metabolic pathways in which peak-neighboring genes were involved. All of the peak-neighboring genes were assigned to different metabolic pathways. Fisher's exact test was used to calculate the significance of gene enrichment in each pathway. In order to screen out the metabolic pathways that were significantly enriched in peak-neighboring genes, the top 15 entries were chosen in the pathway enrich analysis where peak-neighboring genes were significantly enriched as the horizontal axis histogram. The horizontal axis in the histogram was employed to show -log10 (*P* value), and the vertical axis was the pathway name. Meanwhile, the top 25 entries were selected in the pathway enrich analysis in which peak-neighboring genes were significantly enriched as a vertical axis histogram. In the histogram, the horizontal axis showed the pathway name, and the vertical axis represented enrichment. In all charts, red represented significant items, and blue marked nonsignificant items. (b) The results of the pathway enrichment analysis of peak-neighboring genes were presented in a bubble chart. The top 20 pathway entries were selected, in which peak-neighboring genes were significantly enriched for displaying. The ordinate in the figure stood for the pathway entry, and the abscissa indicated the rich factor. The size of the dot in the figure indicated the number of peak-neighbor genes enriched on the pathway entry, and the color represented the significance *P* value of the pathway entry. The smaller the *P* value, the more significant the set. The pathway enrichment degree was measured using the rich factor, *P* value, and the number of genes enriched in this pathway entry. Among them, the rich factor referred to the ratio of the number of peak-neighbor genes enriched in the pathway entry to the number of all genes annotated to the pathway entry. The greater the rich factor, the greater the degree of enrichment.

The results of KEGG enrichment analysis were screened using Fisher's exact test with FDR adjusted *P* value (Figures 7(a) and 7(b)). KEGG results of downregulated genes showed that “NF-kappa B signaling pathway,” “Hedgehog signaling pathway,” and “Hippo signaling pathway” were significantly enriched. Among the upregulated genes, “Hedgehog signaling pathway,” “Inositol phosphate metabolism,” and “TGF-beta signaling pathway” were significantly enriched for signaling pathways except for general cancer processes.

## 3. Discussion

At present, OSCC remains one of the most frequent malignant cancers worldwide, which can rapidly invade the palate, tongue, alveolar ridge, buccal mucosa, and the floor of the mouth. The five-year survival rate of OSCC continues to be low compared to some types of malignancies and is also lower than the expectation of patients. There existed approximately 350,000 new cases and about 170,000 deaths for OSCC in 2018, with the majority of the international OSCC cases in Asia (18, 19). To date, despite the tremendous and innovative advances in the pathophysiology of OSCC (such as oncogenic mechanisms and internal and external environmental influences) and the treatment of different stages (such as surgery, chemotherapy, and radiotherapy), much remains unknown about the exact mechanisms underpinning the occurrence and progression of OSCC. Therefore, it is extremely requisite for the effective detection, therapy, and prognosis monitoring of OSCC to further explore and elucidate molecular mechanisms and develop OSCC-related biomarkers.

The novel regulatory mechanism between ncRNAs and mRNAs, the ceRNA hypothesis presumed by Salmena et al. in 2011, means that the competitive binding of shared miRNAs facilitates the cross-talk between ncRNAs and mRNAs (20–22). In the noncoding region, lncRNAs, as classical ncRNAs, can sponge miRNAs to diminish the expression of miRNAs in downstream intergenic interactions. Accumulating evidence suggests that the lncRNA-miRNA-mRNA ceRNA network assumes a critical role in numerous human cancers (23, 24). In breast cancer (BC), seven lncRNAs associated with BC patients' OS were identified by establishing the lncRNA-miRNA-mRNA ceRNA network (25). Laryngeal cancer (LC) has recently been unraveled to be affected by eight kinds of lncRNAs that can dramatically influence overall survival (26).

M^6^A, as a high-impact RNA modification, holds an immense effect on mRNAs and lncRNAs. Based on the peak frequency distribution, we identified a role of M6A in some genes related to cancer (Figure 4), which also provide a reference for the study of OSCC mechanisms. PICSAR was upregulated in carcinoma tissues and cells, and PICSAR mediated the anticancer potential of miR-125b by downregulating YAP1 (27). It has also been reported that alterations of m^6^A levels of MYC and EGFR are involved in the regulation of cancer pathogenesis and progression (28, 29). Our previous research already confirmed that the overexpression of METTL3, a m^6^A key enzyme, could accelerate cell proliferation, migration, and invasion in vivo and in vitro (14). lncRNA SNHG20 was upregulated in OSCC, and its knockdown inhibited OSCC cell proliferation and tumor growth (30). The enrichment analysis also provided some perspectives for the study of the molecular mechanism of OSCC. It has been reported that METTL3 deletion enhances the activation of NF-*κ*B and STAT3 indirectly, leading to tumor growth and metastasis (31, 32). It has been reported that in OSCC lncRNAs (e.g., ORAOV1) or circRNAs (e.g., circ_0001461) can enhance OSCC invasion and metastasis by targeting NF-*κ*B (33, 34). Besides, activation of hedgehog signaling is associated closely with multidrug resistance (MDR) in OSCC (35, 36). However, further studies are still needed to elucidate the mechanism of mRNAs and lncRNAs in m^6^A modification and the related pathophysiological mechanism of their action in OSCC.

In this research, the epigenetic modification of transcriptome was detected and analyzed in two different groups (HAEC and SCC25) by MeRIP-Seq, followed by the m^6^A-specific peak sequence motif analysis, which uncovered that “GGAC” predominantly arose in the top of the motif results (Figure 2). Additionally, IGV was applied to validate the different enrichment of m^6^A in peak-neighboring genes. The results illustrated that both mRNAs and lncRNAs exhibited the discrepancy of m^6^A (Figure 4). In addition, cancer-related biological processes such as regulation of transcription, miRNAs in cancer, NF-kappa B signaling pathway, and Hedgehog signaling pathway showed significant enrichment (Figures 6 and 7). In conclusion, we can conclude that mRNAs and lncRNAs orchestrated by m^6^A modification may be involved in the lncRNA-miRNA-mRNA ceRNA hypothesis, thus afflicting the pathological mechanism of OSCC. In further studies, we will work on evaluating the biological function and clinical value of m6A in OSCC.

## 4. Conclusion

In summary, a series of m^6^A modifications in mRNAs and lncRNAs were detected by high-throughput sequencing (MeRIP-Seq) in SCC25 and HAEC, which preliminarily explored some key clues of molecular mechanistic investigations in OSCC-related pathways with significant potential values. These clues can direct further research on the specific mechanisms of mRNAs and lncRNAs and their m^6^A in OSCC.

## 5. Materials and Methods

### 5.1. Cell Culture

SCC25 was provided by American Type Culture Collection (Manassas, VA, USA), and human aortic endothelial cells (HAECs) were purchased from Human Aortic Endothelial Cells at the Institute of Biochemistry and Cell Biology of the Chinese Academy of Sciences (Shanghai, China). Cells were cultured in Dulbecco's modified Eagle's medium (Gibco, Carlsbad, CA, USA) encompassing 100 U/mL penicillin, 100 mg/mL streptomycin, and 10% fetal bovine serum.

### 5.2. Methylated RNA Immunoprecipitation Sequencing (MeRIP-Seq)

MeRIP-Seq service was obtained from Shanghai Jiayin Biotechnology Co., Ltd. (Shanghai, China). MeRIP-Seq was invented to detect and analyze the epigenetic modification of transcriptomes in cells. Each group consisted of two kinds of sample RNAs that were IP samples and input control samples, which were fragmented to ~100 nucleotides. Thereafter, the RNA fragments were immunoprecipitated using 30 *μ*L protein A magnetic beads (10002D; Thermo Fisher Scientific), 30 *μ*L protein G magnetic beads (10004D; Thermo Fisher Scientific), and affinity-purified anti-m^6^A polyclonal antibodies (ABE572, Millipore, Darmstadt, Germany). After substantial rinsing, the m^6^A fragments were boosted. Next, RNA fragments were washed using RNeasy Mini Kit (74106; QIAGEN, Hilden, Germany), centrifuged in an RNeasy MiniElute spin column (QIAGEN), and eluted using ultrapure H_2_O to harvest RNA with m^6^A enrichment. Then, the RNA-seq libraries are prepared. Clustered libraries were loaded onto a reagent cartridge and forwarded for sequencing runs on the Illumina NovaSeq 6000 platform.

### 5.3. Bioinformatic Analysis

Pattern enrichment analysis of the identified m6A peaks was performed with HOMER. The metagenic m6A distribution was characterized using the MetaPlotR in R. Differentially methylated sites (*P* < 0.05) were identified using diffReps with fold change ≥ 2. Genes of interest were visualized in the IGV (Integrative Genomics Viewer) software. Enrichment analysis of differentially methylated protein-coding genes was performed using GO (http://www.geneontology.org) and the Kyoto Encyclopedia of Genes and Genomes (KEGG) database (http://www.genome.jp/kegg) (Figure 1).

## Data Availability

The raw MeRIP-Seq reveals the m6A modification mapping of mRNA and lncRNA in OSCC are available in the NCBI Gene Expression Omnibus (GEO): accession number GSE197457, [https://www.ncbi.nlm.nih.gov/geo/query/acc.cgi?acc=GSE197457], token: [onmvyokwbpehtgf].

## Abbreviations

m6A: N6-methyladenosine
mRNA: Messenger RNA
ncRNA: Noncoding RNA
lncRNA: Long noncoding RNA
ceRNA: Endogenous RNA
MeRIP-Seq: Methylated RNA immunoprecipitation with next generation sequencing
3′UTRs: 3′ untranslated regions
5′UTR: 5′ untranslated regions
CDS: Sequence coding for amino acids in protein
GO: Gene Ontology
pri-miRNA: Primary miRNA
rRNA: Ribosomal RNA
tRNA: Transfer RNA
eRNA: Enhancer RNA
m5C: RNA 5­methylcytosine
m1A: N1­methyladenosine
m7G: 7­Methylguanosine
U-tail: Pseudouridine, uridylation
CRC: Colorectal cancer
HSPCs: Hematopoietic stem/progenitor cells
OSCC: Oral squamous cell carcinoma
IP: Immunoprecipitation
SCC25: Human tongue phosphorous carcinoma cells
HAEC: Human aortic endothelial cells
IGV: Integrative genomics viewer
BP: Biological process
MF: Molecular function
CC: Cellular component
FDR: False discovery rate
BC: Breast cancer
LC: Laryngeal cancer
CSCC: Cutaneous squamous cell carcinoma.
